# Percutaneous Vertebroplasty for Painful Osteoporotic Vertebral Compression Fractures: A Retrospective Comparative Study of a Negative Pressure‐Guided Technique

**DOI:** 10.1111/os.70432

**Published:** 2026-09-17

**Authors:** Botao Zhang, Zihan Wang, Shibing Zhang, Zhipeng Xi, Xin Chen, Qingyuan Ru, Ke Wu, Rongrong Deng, Xin Liu, Ran Kang

**Affiliations:** ^1^ Department of Spine Surgery Affiliated Hospital of Integrated Traditional Chinese and Western Medicine, Nanjing University of Chinese Medicine Nanjing Jiangsu Province China; ^2^ Suzhou Hospital, Xiyuan Hospital of China Academy of Chinese Medical Sciences (Suzhou TCM Hospital) Suzhou Jiangsu Province China; ^3^ Wuxi Hospital Affiliated to Nanjing University of Chinese Medicine Wuxi Jiangsu Province China; ^4^ Department of Orthopaedics The Second Affiliated Hospital of Nanjing University of Chinese Medicine Nanjing Jiangsu Province China

**Keywords:** bone cement distribution, negative pressure guided percutaneous vertebroplasty, vertebral compression fractures

## Abstract

**Objective:**

To conduct a preliminary evaluation of the feasibility, safety, and radiographic characteristics of a negative pressure‐guided percutaneous vertebroplasty (PVP) technique. The primary focus of this study is on the distribution of bone cement and perioperative hemodynamic changes, with the overarching aim of mitigating the common perioperative risks associated with conventional PVP.

**Methods:**

In this retrospective comparative study, patients with vertebral compression fractures underwent either conventional percutaneous vertebroplasty (Normal PVP) or negative pressure‐guided percutaneous vertebroplasty (Guided PVP). In the Normal PVP group, bone cement was injected via bilateral pedicles. In the Guided PVP group, cement was injected through a unilateral pedicle while negative pressure suction was applied through the contralateral pedicle. The following parameters were assessed: injected cement volume, perioperative vital signs, anterior vertebral height, local kyphosis angle, visual analogue scale (VAS), Oswestry Disability Index (ODI), and postoperative adverse events. A total of 60 patients were included, with 30 patients in each group.

**Results:**

The negative pressure‐guided technique was associated with a more homogeneous bone cement distribution, with a distribution index of 1.10 ± 0.12 in the Guided PVP group compared with 0.94 ± 0.18 in the Normal PVP group (*p* < 0.001). Smaller perioperative fluctuations in mean arterial pressure were observed in the Guided PVP group at 6 s, 3 min, and 5 min after cement injection (*p* < 0.05). At 3 months postoperatively, anterior vertebral height preservation was greater in the Guided PVP group than in the Normal PVP group (*p* = 0.017).

**Conclusion:**

Both groups demonstrated significant pain relief and functional improvement, with no statistically significant differences between groups. Negative pressure‐guided PVP appears to be a feasible and safe technique that may promote more favorable bone cement distribution and attenuate perioperative hemodynamic fluctuations. However, given the retrospective design and limited sample size, these findings should be interpreted as exploratory, and further prospective studies are warranted to clarify its clinical advantages.

## Graphical abstract

In 60 patients with vertebral compression fractures, negative‐pressure‐guided vertebroplasty (NPVP) promoted more uniform cement distribution and reduced cement leakage compared with conventional PVP, resulting in greater perioperative safety and improved radiographic and clinical outcomes at 3 months.

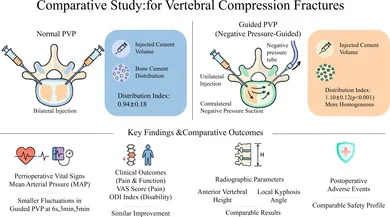

**Abbreviations**
ODI: oswestry disability index
PMMA: polymethyl methacrylate
PVP: vertebroplasty
VAS: visual analogue scale

## 1 Introduction

With the global demographic shift toward an aging population, vertebral compression fractures have become one of the most prevalent musculoskeletal disorders in older adults, posing a substantial burden on healthcare systems and significantly impairing patients' quality of life. Epidemiological studies have reported that the incidence of vertebral compression fractures may reach 30%–50% in individuals over the age of 50 [1]. Percutaneous vertebroplasty (PVP), a minimally invasive procedure involving the injection of polymethyl methacrylate (PMMA) bone cement into the fractured vertebral body, has been widely adopted for the treatment of vertebral compression fractures. This technique can rapidly stabilize the fractured vertebra, restore partial vertebral height, alleviate pain, and facilitate early mobilization [2, 3]. Owing to these advantages, PVP has become one of the most commonly performed interventions in clinical practice.

Despite its clinical efficacy, concerns regarding the safety of PVP persist, particularly with respect to cement leakage and perioperative complications. Previous studies have reported cement leakage rates ranging from 20% to over 30%, although many cases are asymptomatic [4]. When leakage occurs into critical anatomical structures, however, it may lead to severe and potentially life‐threatening complications. Cement extravasation into the spinal canal may cause compression of the spinal cord or cauda equina, resulting in neurological deficits [5]. Leakage into the intervertebral foramen can induce nerve root compression and radicular symptoms [6], while migration of cement into the vertebral venous system may lead to pulmonary or other organ embolisms [7]. These complications underscore the importance of optimizing cement delivery techniques to improve procedural safety.

In response to these risks, previous efforts have focused on both modifying bone cement materials and refining surgical techniques. Alternative cement formulations, such as calcium sulfate‐based bone cements, have been investigated because of their favorable biocompatibility and osteoconductive properties [8]. However, these materials often exhibit limitations, including inferior mechanical strength, suboptimal injectability, and accelerated degradation, which restrict their widespread clinical application as substitutes for PMMA [9]. From a technical perspective, balloon‐assisted kyphoplasty has been introduced to reduce injection pressure and cement leakage by creating an intravertebral cavity prior to cement injection. Nevertheless, cavity formation may promote localized cement aggregation rather than homogeneous distribution, potentially compromising mechanical stability and predisposing to cement loosening or displacement after surgery [10].

From a biomechanical standpoint, cement leakage during conventional PVP is thought to be closely associated with elevated intravertebral pressure generated during cement injection. High syringe pressure increases intravertebral pressure relative to adjacent venous structures and surrounding soft tissues, thereby facilitating cement extrusion through cortical defects or venous channels. Based on this rationale, strategies aimed at modulating intravertebral pressure during cement injection may help improve cement distribution and reduce leakage risk.

Therefore, this retrospective exploratory study had three objectives.

- To evaluate the feasibility and radiographic characteristics of the Guided PVP technique—including bone cement distribution pattern, cement leakage rate, and vertebral height restoration—compared with Normal PVP. More homogeneous cement dispersion through controlled intravertebral pressure modulation, potentially reducing leakage. Creation of a contralateral negative‐pressure gradient, enabling unilateral injection with bilateral cement distribution. Prospective studies with extended follow‐up are needed to confirm whether improved distribution translates into long‐term vertebral stability and reduced risk of adjacent vertebral fractures.
- To compare short‐term clinical outcomes between Guided PVP and Normal PVP, including pain relief and functional improvement. Demonstration of noninferior or comparable early symptom relief. Exploratory design with retrospective data, providing preliminary rather than definitive comparative evidence.
- To assess perioperative physiological responses—specifically hemodynamic fluctuations and their potential association with reduced MMA monomer exposure—thereby exploring the proposed physiological benefit of the Guided PVP technique. Potential mitigation of MMA‐induced hemodynamic instability, a safety concern not systematically addressed in Normal PVP. Exploratory correlation between arterial MMA levels (measured in a small subset) and real‐time hemodynamic monitoring. Adequately powered pharmacokinetic studies with serial sampling are required to establish causality and determine clinical relevance, particularly in cardiopulmonary comorbidities.

In the present study, we applied a modified vertebroplasty technique, referred to as negative pressure‐guided percutaneous vertebroplasty (Guided PVP). This technique involves unilateral cement injection combined with controlled negative pressure suction through the contralateral pedicle, with the aim of creating a relative intravertebral pressure gradient during cement delivery. By reducing intravertebral pressure relative to the syringe and surrounding tissues, this approach is hypothesized to facilitate more homogeneous cement distribution and potentially attenuate abrupt perioperative hemodynamic fluctuations.

Although a similar aspiration‐assisted technique was previously described in a small clinical series by Hsin‐Tzu Lu et al. [11], comprehensive radiographic evaluation and clinical characterization of this approach remain limited. Therefore, the present retrospective comparative study was designed as an exploratory investigation to assess the feasibility, radiographic characteristics, and short‐term clinical outcomes of Guided PVP compared with conventional PVP. We specifically focused on bone cement distribution, perioperative hemodynamic changes, vertebral height maintenance, and early clinical outcomes, with the goal of providing preliminary data to inform future prospective studies.

## 2 Methods

### 2.1 Trial Design

This retrospective comparative study was approved by the Institutional Review Board of Jiangsu Province Hospital of Integrated Chinese and Western Medicine (Ethics Number: 2023‐LWKYZ‐074). Due to the retrospective nature of the study, the requirement for trial registration was waived. Written informed consent for treatment was obtained from all patients, and additional consent was obtained for the use of clinical data and postoperative imaging for research purposes when applicable. Patients diagnosed with painful osteoporotic vertebral compression fractures who underwent PVP at the Affiliated Hospital of Integrated Traditional Chinese and Western Medicine between June 2023 and June 2024 were retrospectively reviewed.

### 2.2 Diagnostic Criteria

According to the ACR Appropriateness Criteria Management of Vertebral Compression Fractures [12], the physical examination revealed acute or chronic persistent lumbago and backache, which may be accompanied by radiating pain in the thoracic ribs and positive pressure pain over the spinous processes of the affected vertebrae. Additionally, percussion pain and limited spinal mobility were observed, with or without thoracic and lumbar kyphosis or scoliosis deformities. X‐rays disclosed “wedge‐shaped” or “biconcave” vertebrae, characterized by sparse trabeculae and the occasional presence of the “vacuum sign.” Magnetic resonance imaging confirmed recent fractures by demonstrating high signal intensity in both T2‐weighted and fat‐suppressed sequences, pinpointing the exact location within the vertebra and identifying occult fractures.

### 2.3 Inclusion and Exclusion Criteria

Our study cohort consists of patients aged 50–95 years with vertebral compression fractures diagnosed within the previous 30 days, excluding those with surgical contraindications. Eligibility criteria include obtaining signed informed consent, possessing complete case data, having reliable contact information, demonstrating the ability to comply with regular follow‐ups, having up to two fractured vertebrae, undergoing ineffective conservative treatment, and maintaining a stable condition with comprehensive data over a minimum 3‐month follow‐up period.

Patients are excluded from the study if they are present with pathological vertebral compression due to vertebral tumors or myeloma, intravertebral canal lesions, or radicular pain unrelated to vertebral collapse. Exclusion criteria also include severe organ insufficiency or hematopoietic/coagulopathic dysfunction, a history of psychiatric disorders or dementia that could compromise cooperation during examinations, or an inability to comply with examination requirements. Furthermore, individuals who undergo long‐term glucocorticoid therapy, with systemic or puncture site infections, those who have experienced pain reduction following conservative treatment, and those with coagulation disorders or osteomyelitis are ineligible for participation. Patients with a fracture of the anterior vertebral body were excluded from the study.

### 2.4 Negative Pressure Guided Technique for Vertebral Fracture Models

Six eligible patients with vertebral compression fractures were selected for this study. Computed tomography data of their vertebrae were obtained to construct 3D‐printed fracture models, retaining only the cortical parts and ensuring that the vertebrae formed sealed cavities. To emulate the internal architecture of compromised vertebrae, sponges were utilized and saturated in physiological saline at 37°C before being positioned within the enclosed cavities of the vertebral models. Identical surgical instruments (Shandong Guanlong) were used for the punctures, and PMMA bone cement (Tecres S.P.A., Italy) was selected as the filling material. Three models were designated for Normal PVP and three for Guided PVP. In the Guided PVP group, aspiration needles were placed in the anterior, middle, and posterior thirds of the vertebrae. Negative pressure was titrated, commencing at 0 kPa. Bone cement was administered via an injection needle, initially introduced into the anterior third of the vertebrae. This needle was incrementally retracted while concurrently advancing the cement, culminating in the middle third of the vertebrae. The amount of bone cement injected was carefully recorded, and subsequent sectioning of the models allowed for analysis of bone cement distribution within the vertebrae (Figure 1).

**FIGURE 1:**
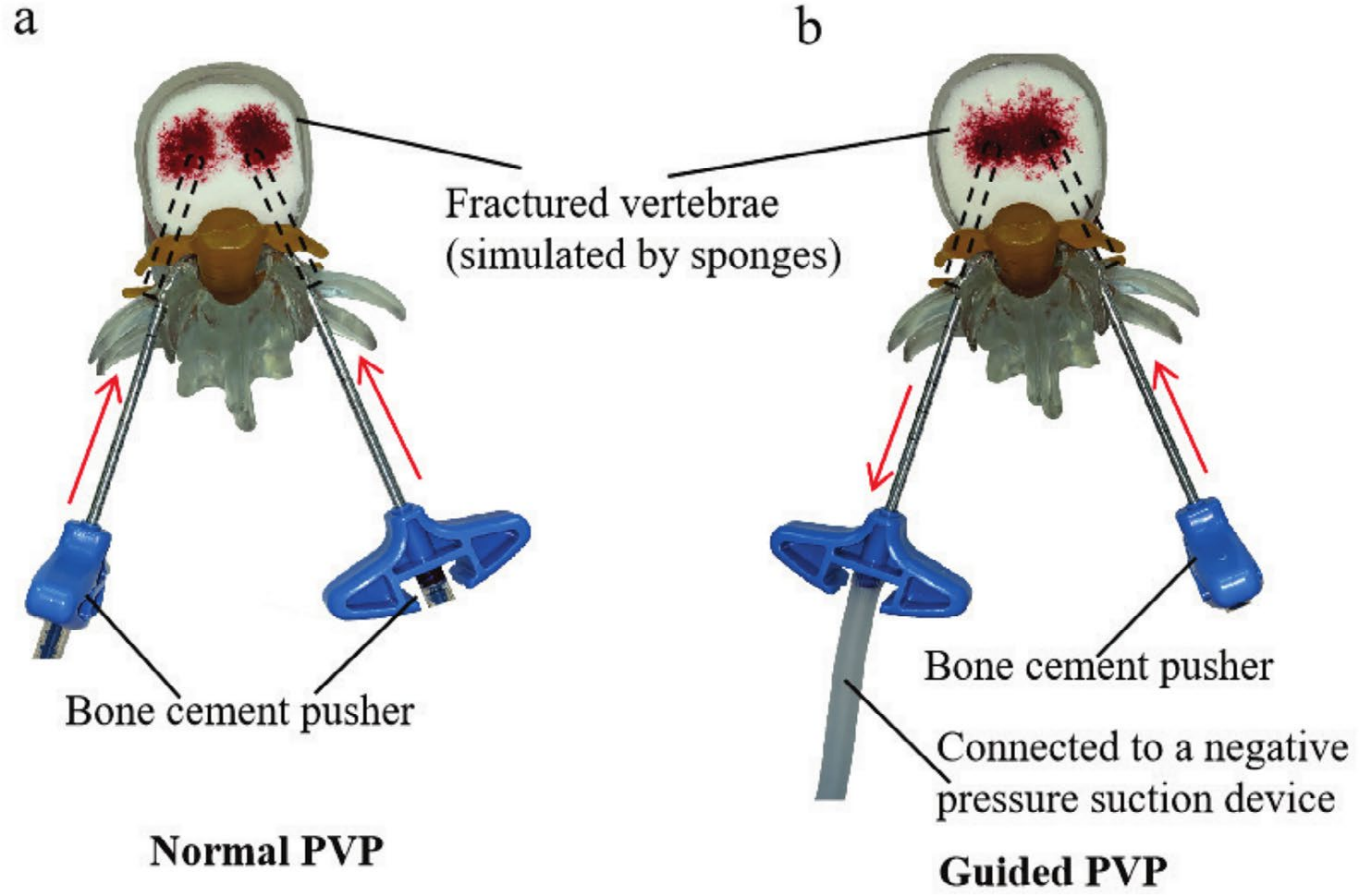
In vitro model of vertebral compression fractures. (a) Model of normal PVP; (b) Model of guided PVP.

It was found that a negative pressure range of −40 to −60 kPa was appropriate; pressures too low made it difficult to establish negative pressure within the vertebra, while pressures too high led to insufficient bone cement on the pushing side. Optimal cement distribution was achieved when the suction device was positioned in the middle third of the vertebrae, a critical parameter for ensuring effective vertebroplasty outcomes. This negative pressure parameter will guide subsequent trials and will be adjusted according to the actual situation of the participants.

### 2.5 Interventions and Procedures

Prior to undergoing PVP, all patients were required to undergo a comprehensive preoperative laboratory assessment, including complete blood counts, coagulation profiles, and hepatic and renal function tests. The same surgical instruments (Shandong Guanlong) were used for punctures, and PMMA bone cement (Tecres S.P.A., Italy) was selected as the filling material. The Guided PVP group employed Negative Pressure Guided PVP, as described in the following procedures.

Local infiltration anesthesia was administered to all patients. Patients were positioned prone on the lumbar bridge of the operating table, and fluoroscopic imaging using a C‐arm machine was employed to identify the target vertebrae and outline their pedicles and transverse processes. Surgical site preparation began with sterilization of the operative fields using an iodophor solution. Percutaneous needle entry points were established bilaterally, 3 mm lateral to the pedicle projections, avoiding the medial wall while advancing toward the mid‐third of the vertebrae. Bone cement injection was guided by fluoroscopy and assisted by a negative pressure suction device, with settings ranging from −40 to −60 kPa to minimize the risk of leakage (Figure 2).

**FIGURE 2:**
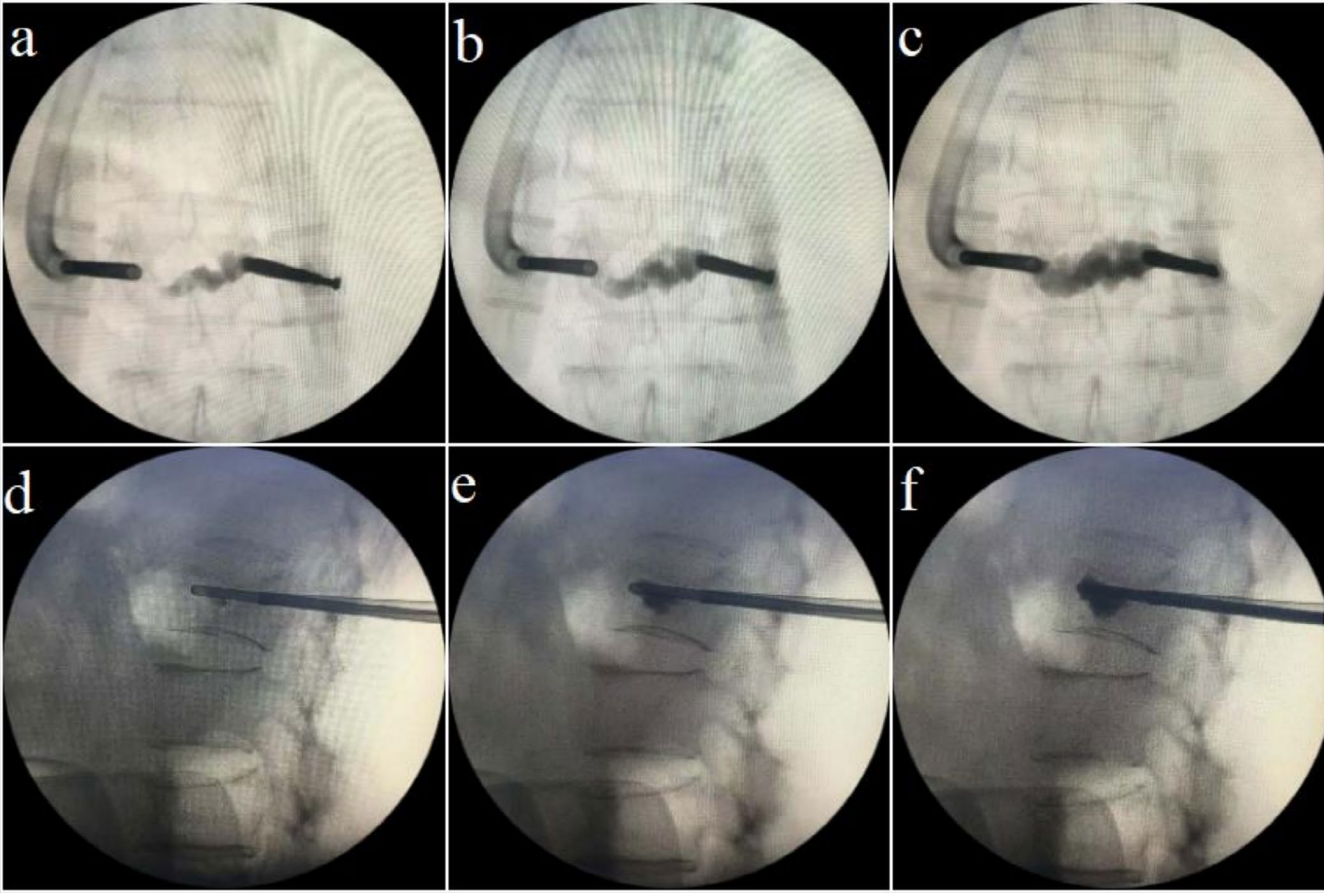
Surgical procedure of guided PVP. (a–c) intraoperative fluoroscopy (crown position). (d–f) intraoperative fluoroscopy (sagittal position). The radiograph (X‐ray) was taken on October 23, 2023.

The injection needle was incrementally retracted while concurrently advancing the cement, culminating in the middle third of the vertebrae. Postoperatively, the incision was dressed with a sterile covering. In accordance with the predefined protocol, patients were permitted to ambulate within the ward, supported by a lumbar brace, approximately 6–8 h after surgery, imperative to ensure that the bone cement is thoroughly anchored to the fractured vertebrae (In mainland China, such vertebral fractures require inpatient surgery rather than outpatient procedures).

The Normal PVP group followed the same protocol as described above, except for attaching bone cement‐filled syringes to the bilateral puncture needle tips. The utilization of bilateral puncture as opposed to unilateral puncture is intended to ensure optimal diffusion and support of bone cement within the vertebrae, thereby ensuring no significant difference in bone cement injection volume between the two groups. All surgical procedures are carried out autonomously by the same chief physician.

In our study, no persistent bleeding was observed during negative pressure application. Only minimal bleeding (< 10 mL) occurred within the first 3 s of aspiration in all patients. We had a predefined safety protocol: if continuous intravertebral bleeding occurred, we would stop the procedure, withdraw the patient from the study, and, in consultation with an anesthesiologist, decide to either abort the surgery, complete conventional vertebroplasty, or perform unilateral puncture on the unaffected side. This protocol ensured patient safety throughout.

### 2.6 Observation Indicators

Safety parameters were stringently monitored throughout the treatment phase and the subsequent follow‐up period. Intraoperatively, a small volume of arterial blood was collected from three individuals in each group immediately following the injection for qualitative analysis of methyl methacrylate (MMA) in arterial serum using gas chromatography–mass spectrometry (GC–MS). This analysis detected MMA in the Normal PVP group, whereas no MMA was identified in the other group (Figure 3).

**FIGURE 3:**
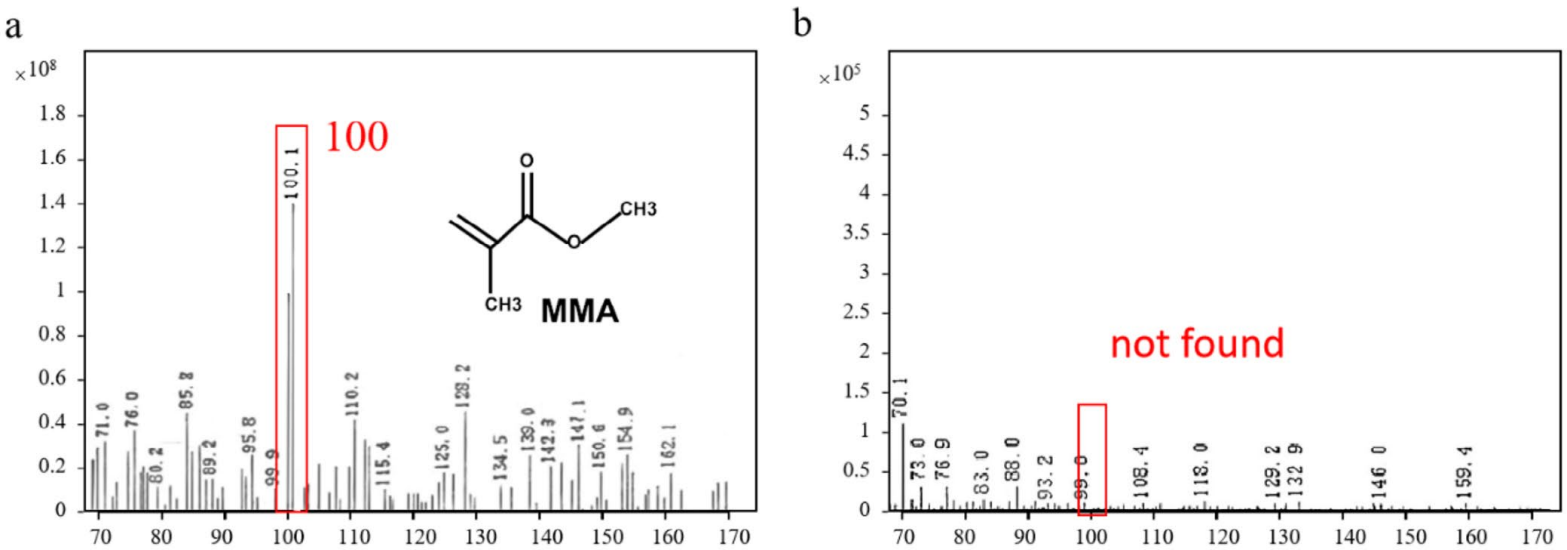
Arterial blood was drawn from patients who had just received Normal PVP or Guided PVP for GC–MS. The molecular weight of MMA is 100. (a) MMA was identified in the arterial blood of patients in the Normal PVP group; (b) MMA was not detected in the arterial blood of patients in the Guided PVP group.

Furthermore, the volume of bone cement injected per participant was meticulously documented. Postoperatively, three‐dimensional models of the bone cement and surgical vertebrae were constructed using Mimics 21.0 software (Materialise, Belgium) and DICOM data derived from computed tomography scans for comprehensive analysis. Subsequent to the surgical procedure, each patient underwent a CT scan of the vertebral bodies. Patients were informed of the potential risks associated with radiation exposure and provided with written informed consent. All CT analyses were performed by the same radiologist. Additionally, vital signs, including blood pressure, heart rate, and blood oxygen saturation, were monitored at specific time points: 30 min preoperatively and at 0.1, 3, 5, and 10 min postinjection. Intraoperative blood pressure and heart rate were monitored using an armband blood pressure monitor, while oxygen saturation was assessed with a pulse oximeter clip. The study meticulously recorded postoperative complications, including cement leakage and adverse reactions such as asthma and pulmonary embolism, with follow‐up evaluations conducted within 3 days, 1 month, and 3 months.

Moreover, the Anterior Vertebral Height (AVH) and Local Kyphosis Angle (LKA) were measured using X‐ray imaging at 3 days, 1 month, and 3 months postoperatively, with careful monitoring for cement leakage. Additionally, patients were evaluated both preoperatively and at 3 days, 1 month, and 3 months postoperatively using the Visual Analogue Scale (VAS) for back pain and the Oswestry Disability Index (ODI) for functional impairment.

### 2.7 Statistical Analysis

Statistical analyses were performed using SPSS version 23.0 (IBM Corporation, USA). Continuous data are presented as mean ± standard deviation. Independent samples *t*‐tests were used to compare continuous variables in general information, and Welch's *t*‐tests were employed when variances were heterogeneous. Categorical data were compared using chi‐square tests. A repeated measures two‐way ANOVA was conducted for within‐group and between‐group analyses of the outcomes. A *p* value of less than 0.05 was considered statistically significant.

## 3 Results

### 3.1 Participants

A total of 66 participants were enrolled between June 2023 and June 2024, comprising 34 individuals in the Normal PVP group and 32 in the Guided PVP group. During the injection, four participants in the Normal PVP group experienced hypotension, prompting the anesthesiology team to administer vasopressor agents. In the Guided PVP group, two individuals experienced intolerable pain associated with the suctioning process. Ultimately, six participants withdrew from the study, resulting in 60 patients successfully completing the study protocol (Figure 4).

**FIGURE 4:**
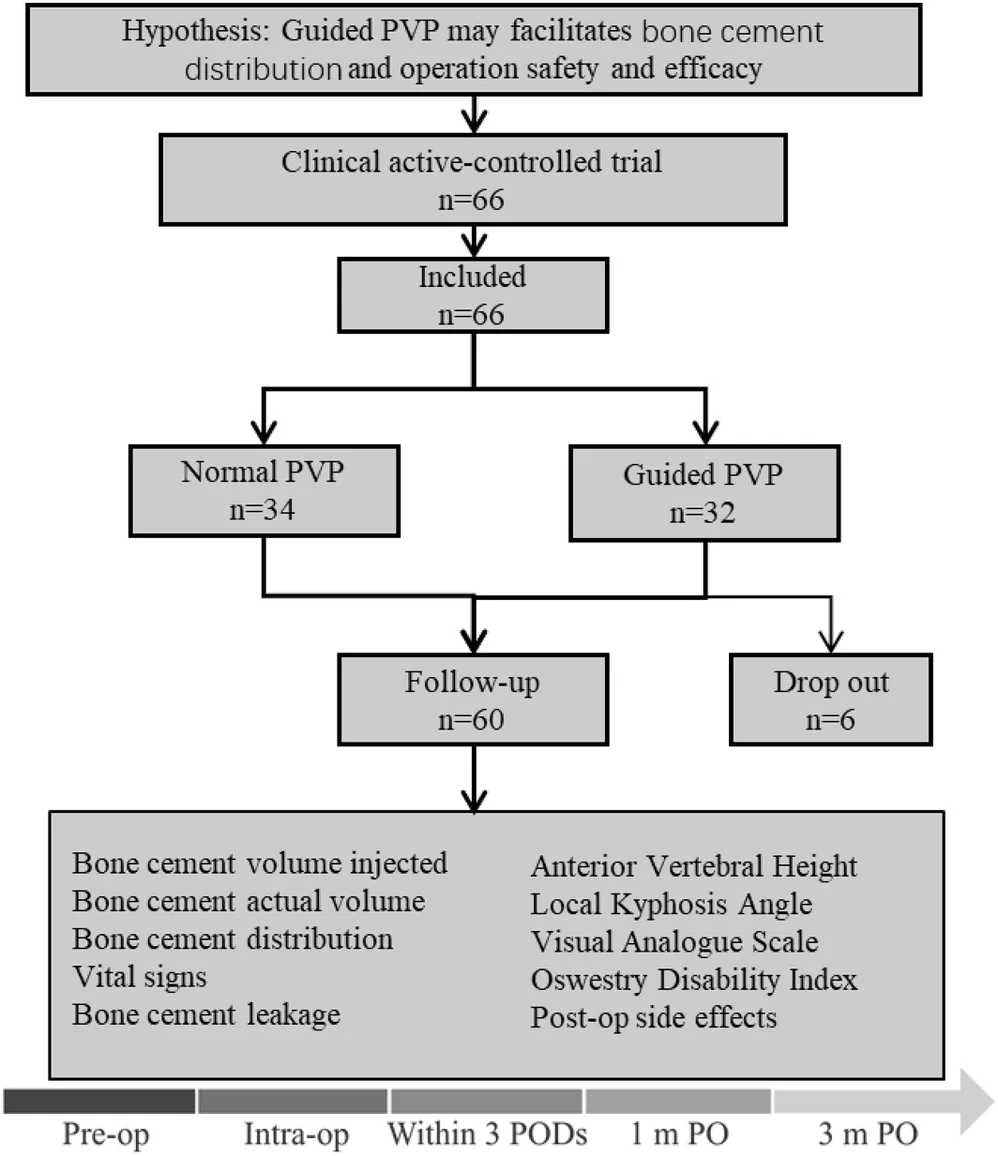
Flow diagram of patient enrollment and study timeline. Sixty patients with vertebral compression fractures were assigned to Guided PVP or conventional PVP. Hypotension during injection occurred in four Normal PVP participants (requiring vasopressors) and suction‐induced intolerable pain in two Guided PVP participants.

The Normal PVP group consisted of 7 males and 23 females, aged between 57 and 88 years, with a mean treatment duration of 10.7 ± 8.2 days. The Guided PVP group included 3 males and 27 females, aged between 56 and 95 years, with a mean treatment duration of 9 days. The average surgical time for the Guided PVP group was 55 ± 16.2 min, compared to 49 ± 7.9 min for the Normal PVP group. There were no significant differences in the demographic and baseline characteristics between the two groups. All participants received standard pharmacological treatment and were followed for at least 3 months. Detailed demographic data and procedural specifics are presented in Table 1.

**TABLE 1:** The demographic data of 60 fractured vertebrae.

Variable categories	Normal PVP (*n* = 30)		Guided PVP (*n* = 30)		*p*
	*n*	Mean ± SD	*n*	Mean ± SD	
Gender					
Male	7		3		0.166
Female	23		27		
Age (years)		75.6 ± 7.5		75.1 ± 9.7	0.823
Injury duration (days)		10.7 ± 8.2		9.2 ± 4.6	0.384
Operation duration (min)		55 ± 16.2		49 ± 7.9	0.273
Centrum					
T8	0		1		0.076
T9	0		1		
T10	1		2		
T11	2		3		
T12	9		10		
L1	8		9		
L2	5		4		
L3	4		0		
L4	1		0		
Mean follow‐up duration (days)	90		90		

### 3.2 Safety Indicators

The volume of bone cement administered to each participant was meticulously documented throughout the surgical procedure. Postoperatively, computed tomography (CT) scans were conducted on the operated vertebrae to quantitatively assess the volume of bone cement within the treated vertebrae. No significant differences were observed between the two groups regarding the volume of bone cement injected and the actual volume of bone cement. The degree of bone cement distribution was calculated as the actual volume divided by the volume injected. The Guided PVP group exhibited a significantly higher distribution than the Normal PVP group. The mean distribution values were 1.10 ± 0.12 for the Guided PVP group and 0.94 ± 0.18 for the Normal PVP group, with the difference reaching statistical significance (*p* = 0.0001) (Figure 5).

**FIGURE 5:**
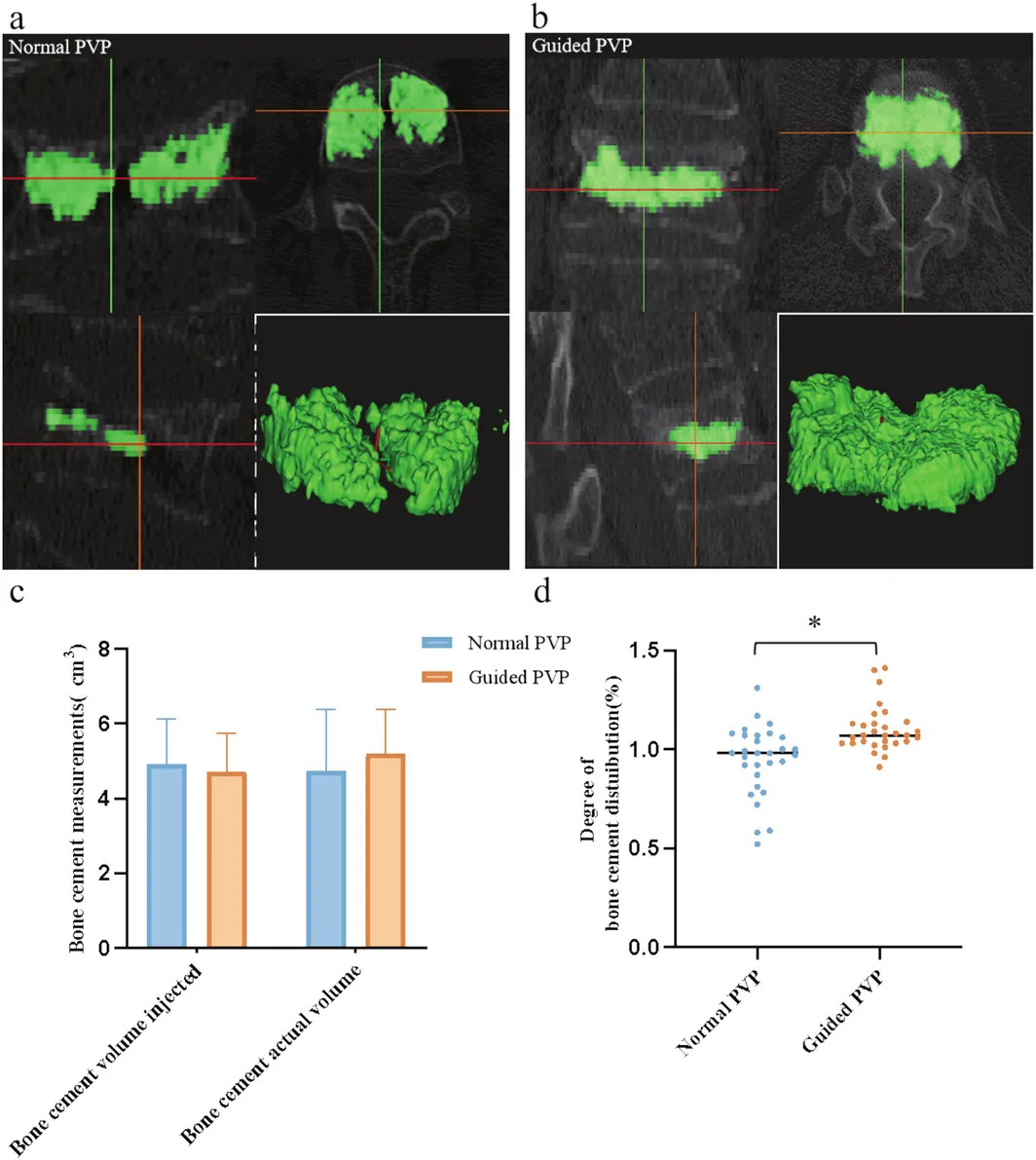
Distribution characteristics of bone cement. (a, b) Three‐dimensional reconstruction of postoperative bone cement distribution (Guided PVP vs. Normal PVP); (c) Injected bone cement volume and actual bone cement volume; (d) Degree of bone cement distribution. The degree of bone cement distribution was defined as the ratio of actual bone cement volume to injected bone cement volume. *p < 0.05.

Intraoperative monitoring of vital signs, as depicted in Figure 6, indicated that the Normal PVP group experienced a significantly greater reduction in mean arterial pressure (MAP) at specific time points compared to the Guided PVP group: from 30 min preoperative to 0.1 min postinjection (22.6 ± 10.41 mm Hg vs. 12.57 ± 9.12 mm Hg, *p* = 0.0002), from 30 min preoperative to 3 min postinjection (14.6 ± 9.88 mm Hg vs. 6.3 ± 8.82 mm Hg, *p* = 0.001), and from 30 min preoperative to 5 min postinjection (10.3 ± 11.01 mm Hg vs. 2.13 ± 9.97 mm Hg, *p* = 0.004). No statistically significant differences were observed from 30 min preoperatively to 10 min postinjection. MAP was calculated as Diastolic Pressure + (1/3) × (Systolic Pressure—Diastolic Pressure). Furthermore, there were no significant differences in intraoperative heart rate and blood oxygen saturation between the two groups (Figure 6).

**FIGURE 6:**
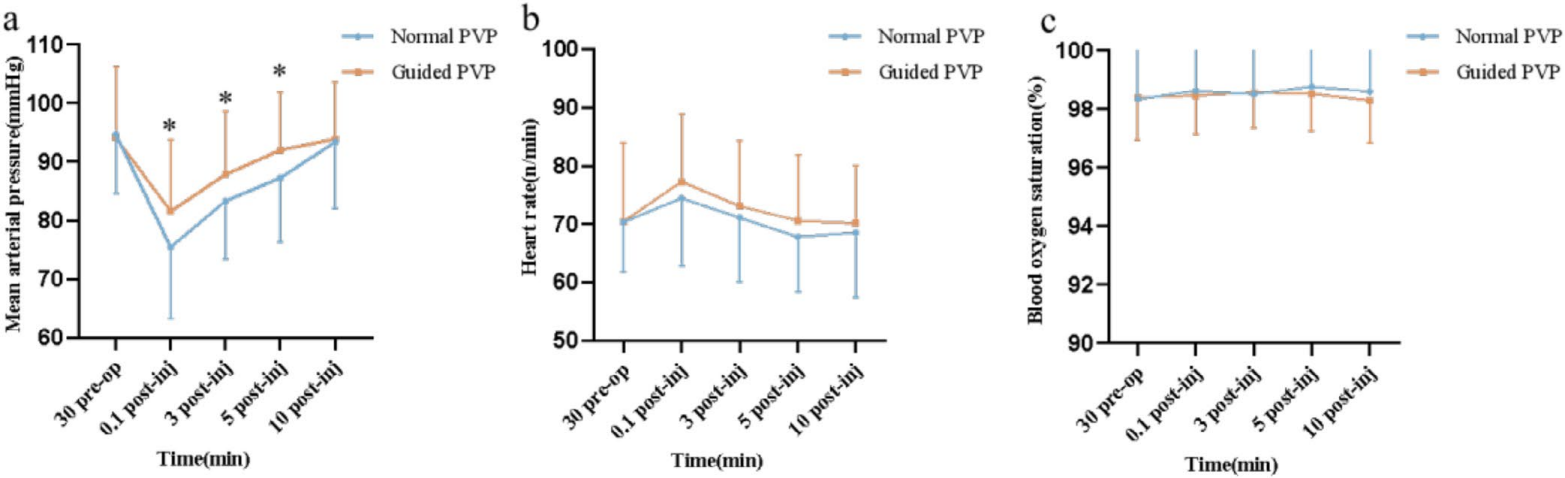
Intraoperative vital sign dynamics during PVP. (a) Mean arterial pressure (MAP); (b) Heart rate; (c) Blood oxygen. *p < 0.05. Differences in MAP were calculated as the change from the preoperative value measured 30 min before surgery and compared between groups.

In both groups, mean arterial pressure significantly decreased and heart rate significantly increased at 0.1 min following the initial push, relative to values measured 30 min preoperatively (*p* < 0.05), while blood oxygen saturation remained stable. A notable rebound in mean arterial pressure and a partial decrease in heart rate was observed at 3 min postinjection (*p* < 0.05). Over the next 7 min, mean arterial pressure gradually increased and heart rate decreased, returning to preoperative levels by the 10‐min mark. Blood oxygen saturation showed minimal variation throughout the procedure.

Postoperative imaging identified one case of paravertebral soft tissue leakage and one of perivertebral leakage in the Normal PVP group, along with four cases of intradiscal leakage. In stark contrast, the Guided PVP group showed only a single instance of intradiscal leakage (*p* < 0.05). At the 6‐month follow‐up, no additional procedure‐related adverse events, such as asthma or asymptomatic pulmonary embolism, were observed in either group. Furthermore, no new compression fractures in adjacent vertebrae were reported in both groups during the 6‐month follow‐up period.

### 3.3 Radiological Assessment

At the three‐month postoperative follow‐up, the AVH in the Guided PVP group was more stable, with smaller changes, than that in the Normal PVP group, as compared to initial assessments conducted within the first 3 days postsurgery (0.93 ± 1.10 vs. 1.69 ± 1.30, *p* < 0.05) (Figure 7a,b).

**FIGURE 7:**
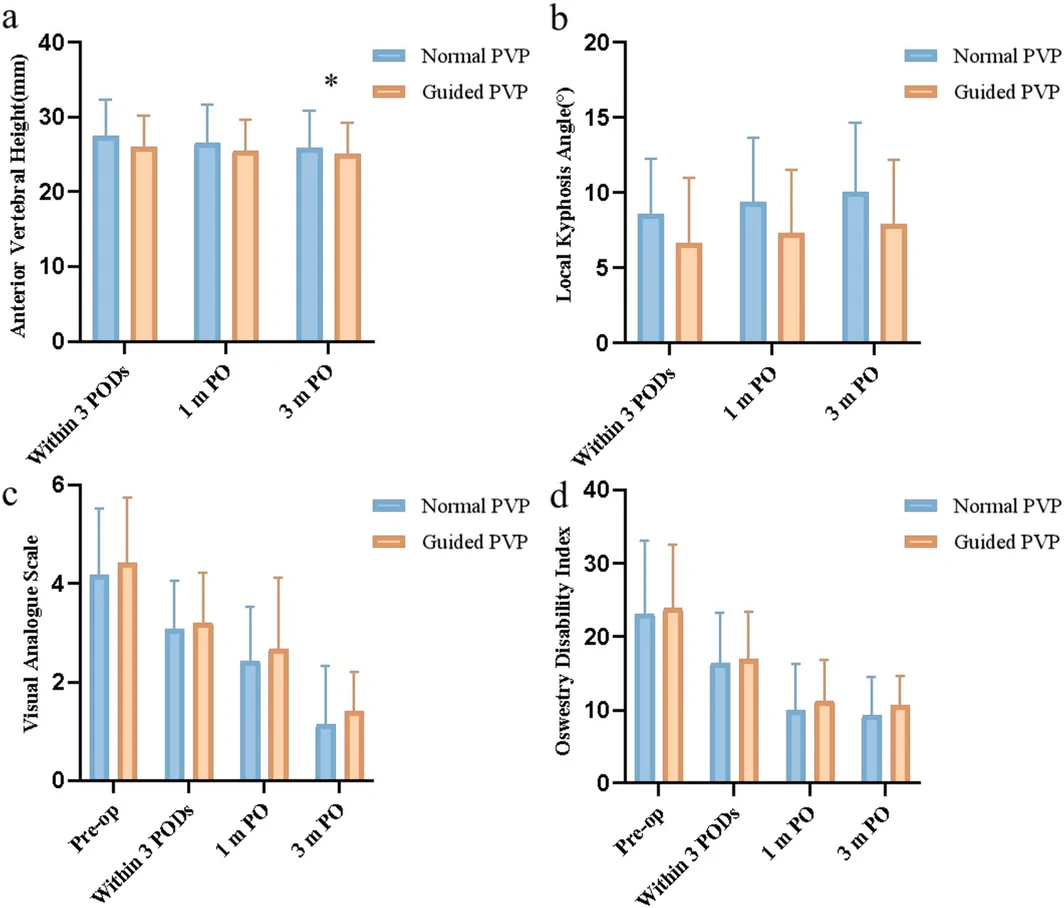
Restoration of vertebral morphology and clinical outcomes. (a) Anterior vertebral height; (b) Local kyphotic angle; (c) Visual analogue scale (VAS); (d) Oswestry Disability Index (ODI). Pre‐op, preoperative; within 3 POD, within the first three postoperative days; 1 m PO, 1 month postoperatively. Differences in anterior vertebral height were calculated as the change from the preoperative baseline value and compared between groups. *p < 0.05.

Furthermore, no significant differences in vertebral heights were observed between the two groups when comparing measurements taken within 3 days postoperatively to those obtained at 1 month postoperatively. The analysis revealed no significant difference in the improvement of the LKA between the two groups (*p* > 0.05).

### 3.4 Function Indicators

Data analysis over a three‐month follow‐up period revealed no significant differences in pain relief or functional recovery between the Normal PVP and Guided PVP groups. Both cohorts demonstrated a substantial reduction in the VAS compared to preoperative levels, with the Normal PVP group showing a decrease from 4.17 to 1.13 and the Guided PVP group from 4.43 to 1.140 by Day 90 (*p* < 0.05). This indicates a significant alleviation of pain in both groups following the procedure. Similarly, the ODI, which measures functional disability, decreased markedly in both groups. The Normal PVP group fell from 23.4 to 9.23, and the Guided PVP group from 23.87 to 10.60 by Day 90 (*p* < 0.05) (Figure 7c,d).

## 4 Discussion

The present study provides exploratory evidence that negative pressure‐guided PVP is feasible and safe, and that its main advantages are not limited to a single radiographic endpoint. Instead, the technique should be understood through three complementary dimensions: biomechanical stability, pain relief and functional recovery, and biosafety during cement delivery. Guided PVP appeared to improve the spatial distribution of bone cement and short‐term preservation of AVH, achieved pain relief comparable to conventional PVP, and was associated with smaller perioperative blood pressure fluctuations and no detectable arterial MMA signal in the limited serum analysis.

These findings suggest that the value of negative pressure guidance lies primarily in controlling the intravertebral environment during cement injection. By using contralateral suction to evacuate marrow, blood, and intravertebral fluid, the technique may create a pressure gradient that facilitates cement permeation while avoiding excessive local pressurization. Because this was a retrospective study with a modest sample size and short follow‐up, the advantages discussed below should be interpreted as hypothesis‐generating and require validation in prospective studies.

### 4.1 Biomechanical Stability

Previous studies have highlighted the importance of cement distribution patterns in determining biomechanical stability following vertebroplasty. Chen *et al*. demonstrated that improved cement dispersion within the vertebral body may reduce stress concentration and lower the risk of adjacent vertebral fractures, thereby contributing to long‐term structural stability [13]. In the present study, contralateral negative pressure suction probably created a more favorable filling environment by evacuating bone marrow and blood from the fractured vertebra and by reducing resistance within the trabecular space. This mechanism may explain why Guided PVP achieved a higher cement distribution index without requiring a greater cement volume. More homogeneous cement interdigitation can enlarge the cement‐bone contact area, improve load sharing across the anterior and middle columns, and reduce focal stress concentration around compact cement masses [14, 15]. The more favorable preservation of AVH observed at short‐term follow‐up further supports a potential biomechanical advantage. Although the present follow‐up was too short to determine whether this translates into fewer adjacent vertebral fractures, these radiographic findings provide a rationale for future long‐term biomechanical and clinical evaluation.

### 4.2 Pain Relief

Both groups achieved marked improvement in VAS and ODI scores, indicating that Guided PVP preserved the established analgesic effect of vertebroplasty. The absence of a significant between‐group difference should not be interpreted as therapeutic inferiority, because pain relief after PVP is mainly driven by rapid stabilization of vertebral microfractures [16], reduction of abnormal micromotion, and thermal or chemical effects during cement polymerization that may decrease intraosseous nociceptive stimulation [17, 18, 19]. Conventional PVP is already highly effective for these mechanisms, which may create a ceiling effect for early pain and functional outcomes. Therefore, the advantage of Guided PVP in this domain is more appropriately described as achieving comparable early pain relief and functional recovery while using a pressure‐guided cement delivery strategy. This point is clinically relevant because a modified technique must first maintain the core therapeutic benefit of PVP before its additional radiographic or safety advantages can be considered meaningful. Longer follow‐up is needed to determine whether improved cement distribution contributes to more durable pain control, better spinal alignment, or reduced recurrence of vertebral collapse [20].

### 4.3 Biosafety

The third advantage of Guided PVP concerns procedural biosafety. Cement leakage remains a major concern during vertebroplasty, particularly when cement enters venous channels, the spinal canal, or the intervertebral foramen [21, 22]. From a pressure‐flow perspective, conventional injections can transiently increase intravertebral pressure and promote cement migration through cortical defects or venous pathways. Negative pressure guidance may counteract this process by providing a controlled outflow route and by reducing abrupt pressure accumulation within the vertebral body. In the present series, clinically evident cement leakage was low in the Guided PVP group, and perioperative mean arterial pressure fluctuations were smaller after cement injection. In addition, MMA was detected in arterial serum samples from the Normal PVP group but not in the Guided PVP group in the limited GC–MS analysis. These observations do not prove reduced systemic toxicity, because the biochemical assessment was qualitative and performed in only a small subset of patients. Nevertheless, they support the hypothesis that controlled cement flow and lower intravertebral pressurization may reduce systemic exposure to cement‐related byproducts and attenuate hemodynamic disturbance. Future studies should include standardized leakage classification, serial MMA measurements, and larger safety datasets to clarify this potential biosafety benefit [23].

We introduce a negative pressure‐guided vertebroplasty technique that facilitates more homogeneous bone cement dispersion via precise intravertebral pressure modulation, while concurrently mitigating acute perioperative hemodynamic fluctuations. Furthermore, this approach reduces the intravascular leakage of MMA, thereby lowering biological toxicity. The superior preservation of AVH observed at short‐term follow‐up lends further support to its potential biomechanical advantages. Nevertheless, owing to methodological constraints and sampling difficulties, blood samples were obtained and analyzed for only three patients, leaving the remainder of the cohort untested. Future investigations will involve intraoperative arterial MMA quantification in a larger patient cohort to substantiate and refine our present conclusions.

Several limitations of this study should be acknowledged. First, the retrospective design and modest sample size limit the strength of causal inference and the generalizability of the results. Second, a small number of patients undergoing Guided PVP experienced discomfort during the aspiration phase, likely related to periosteal irritation, which resulted in procedure discontinuation in two cases. In addition, some patients were unable to tolerate the procedure under local anesthesia, necessitating conversion to general anesthesia [24, 25, 26]. These observations indicate that patient selection and procedural optimization remain important considerations.

A further limitation of this study is the relatively short follow‐up period, which precludes assessment of several important long‐term outcomes. Specifically, we cannot evaluate long‐term vertebrae stability or the incidence of adjacent vertebral fractures. Among these, the potential reduction in adjacent vertebral fractures deserves particular emphasis. The theoretical advantage of negative pressure‐guided vertebroplasty: achieving more controlled cement dispersion with lower intramedullary pressure is that it may reduce the risk of cement leakage and subsequent overload on adjacent motion segments. This biomechanical benefit is precisely the key rationale for the additional procedural effort required by the negative pressure technique. However, validating a genuine reduction in adjacent fracture risk requires extended follow‐up, ideally of at least 12 months, as these events typically manifest in the intermediate to long term rather than immediately postoperatively. Therefore, future prospective studies with follow‐up periods of 1 year or longer are necessary to determine whether the observed biomechanical and physiological advantages translate into sustained clinical benefits, particularly a lower rate of adjacent vertebral fractures.

Finally, the lack of significant differences between groups in pain relief and functional recovery highlights the need for further investigation into the clinical relevance of improved cement distribution. Future prospective studies with larger cohorts, longer follow‐up, and standardized outcome measures are required to clarify the biomechanical and clinical implications of negative pressure‐guided techniques and to define their role in routine clinical practice.

## 5 Conclusion

Negative pressure‐guided PVP is feasible and safe. Its potential advantages can be summarized in three dimensions: improved biomechanical stability through more homogeneous bone cement distribution and better short‐term vertebral height preservation; preserved pain relief and functional recovery comparable to conventional PVP; and possible biosafety benefits reflected by attenuated perioperative hemodynamic fluctuations and limited MMA detection in this exploratory analysis. Normal PVP remains highly effective for pain management, and the present technique should be viewed as an alternative with potential technical and safety advantages. Given the retrospective design, limited sample size, and short follow‐up, these results remain exploratory; prospective studies are needed to further define its incremental clinical value.

## Author Contributions

**Botao Zhang:** writing – original draft, writing – review and editing, project administration, conceptualization, methodology. **Zihan Wang:** conceptualization, methodology, writing – review and editing, writing – original draft. **Shibing Zhang:** validation, investigation, visualization, methodology. **Zhipeng Xi:** methodology, visualization, validation, investigation. **Xin Chen:** software, data curation, formal analysis. **Qingyuan Ru:** software, formal analysis, data curation. **Ke Wu:** investigation, validation, funding acquisition, visualization. **Rongrong Deng:** investigation, validation, visualization, funding acquisition. **Xin Liu:** writing – review and editing, writing – original draft, conceptualization, methodology, project administration, supervision, resources, funding acquisition. **Ran Kang:** conceptualization, methodology, supervision, resources, project administration, writing – review and editing, funding acquisition, writing – original draft.

## Funding

This work was supported by the Chinese Medicine Leading Talent Project of Jiangsu Province (number CZ2023SLJ0304), National Natural Science Foundation of China (number 82302735), Project of Institute of Chinese Medicine, Nanjing University (number ICM2024020), Jiangsu Youth Science and Technology Talent Lifting Project (number JSTJ‐2025‐110), and the Open Research Fund of State Key Laboratory of Digital Medical Engineering (number 2025‐M08).

## Ethics Statement

This retrospective comparative study was approved by the Clinical Ethics Committee of Jiangsu Provincial Hospital of Integrated Traditional Chinese and Western Medicine (Ethics approval number: 2023‐LWKYZ‐074). Due to the retrospective nature of the study, the requirement for trial registration was waived.

## Consent

Written informed consent for treatment was obtained from all patients, and additional consent was obtained for the use of clinical data and postoperative imaging for research purposes when applicable.

## Conflicts of Interest

The authors declare no conflicts of interest.

## Data Availability

The data that support the findings of this study are available on request from the corresponding author. The data are not publicly available due to privacy or ethical restrictions.
